# Respiratory Syncytial Virus Infection in Homeless Populations, Washington, USA

**DOI:** 10.3201/eid2507.181261

**Published:** 2019-07

**Authors:** Jim Boonyaratanakornkit, Seda Ekici, Amalia Magaret, Kathryn Gustafson, Emily Scott, Micaela Haglund, Jane Kuypers, Ronald Pergamit, John Lynch, Helen Y. Chu

**Affiliations:** University of Washington, Seattle, Washington, USA (J. Boonyaratanakornkit, S. Ekici, A. Magaret, K. Gustafson, E. Scott, M. Haglund, J. Kuypers, J. Lynch, H.Y. Chu);; Harborview Medical Center, Seattle (J. Boonyaratanakornkit, R. Pergamit)

**Keywords:** respiratory syncytial virus, RSV, viruses, infection, respiratory infection, influenza virus, influenza, homelessness, homeless persons, homeless populations, chronic obstructive pulmonary disease, COPD, asthma, drug use, Washington, United States

## Abstract

Homelessness has not previously been identified as a risk factor for respiratory syncytial virus (RSV) infection. We conducted an observational study at an urban safety-net hospital in Washington, USA, during 2012–2017. Hospitalized adults with RSV were more likely to be homeless, and several clinical outcome measures were worse with RSV than with influenza.

Respiratory syncytial virus (RSV) is increasingly recognized as a major pathogen in adults and shows a disease burden comparable to that for influenza (*1*). No vaccine is currently available. However, several vaccine candidates against RSV are in clinical trials, and the elderly, those with chronic lung disease, infants, and immunocompromised persons remain priority target populations for prevention efforts (*2*,*3*).

Rates of homelessness are increasing in major urban centers because of lack of affordable housing and slower wage growth (*4*). Homeless persons experience higher rates of illness and death compared with the general population, partly because of infectious diseases from lack of access to sanitation, crowding in shelters, untreated chronic medical conditions, and higher rates of mental health issues and substance use (*5*). Studies have described local outbreaks of rhinovirus and influenza in homeless shelters (*6*). Identification of homeless persons as an at-risk population for severe RSV disease might guide prioritization strategies for RSV vaccines and therapeutics as they become available. We aimed to evaluate risk factors and clinical outcomes of adults hospitalized with RSV infections versus those with influenza in an urban medical center serving a region that had high rates of homelessness.

## The Study

We conducted a retrospective case–control study of adults hospitalized with RSV and influenza at Harborview Medical Center (Seattle, WA, USA) during July 2012–June 2017. This center is an academic tertiary medical center that functions as the safety-net hospital for the Seattle metropolitan area. We identified patients on the basis of laboratory records of specimens containing influenza A/B virus and RSV by using a rapid PCR assay (Focus Diagnostics, https://www.focusdx.com) or the Xpert Xpress Flu/RSV test (Cepheid, http://www.cepheid.com) (Appendix).

**Figure 1:**
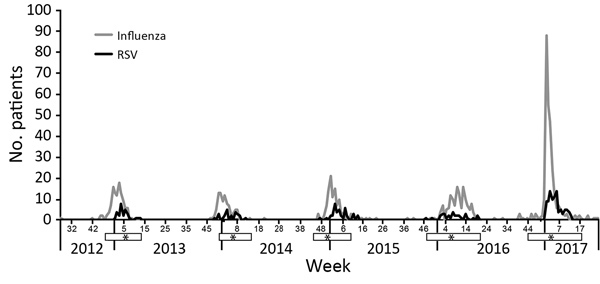
Detection of influenza and RSV in adults hospitalized at Harborview Medical Center, Seattle, WA, USA, July 2012–June 2017. White bars below the x-axis indicate RSV seasons; asterisks indicate weeks when cases of RSV infection peaked, on the basis of Centers for Disease Control and Prevention surveillance data in region 10 (Alaska, Idaho, Washington, and Oregon) (*7**,**8*) during 2012–2017. RSV, respiratory syncytial virus.

**Figure 2:**
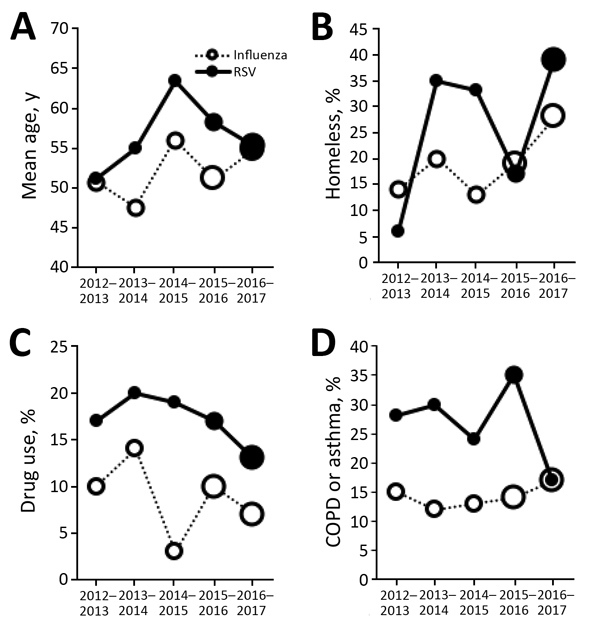
Sociodemographic characteristics of patients hospitalized with RSV infection or influenza across 5 seasons, 2012–2017, Washington, USA. A) Age; B) homelessness; C) drug use; and D) COPD or asthma. Size of each circle indicates number of patients for that data point: small circles indicate <50 patients, medium circles indicate 50–150 patients, and large circles indicate >150 patients. COPD, chronic obstructive pulmonary disease; RSV, respiratory syncytial virus.

A total of 865 patients were hospitalized with RSV infection (n = 157) or influenza A/B (n = 708) during July 2012–June 2017 (Table 1; Figure 1). We showed by multivariable analysis of risk factors for hospitalization with RSV infection versus influenza that older age, homelessness, having chronic obstructive pulmonary disease (COPD) or asthma, and drug use were associated with an increased odds ratio (OR) for RSV hospitalization compared with influenza (Table 1; Figure 2). Drug use showed a correlation with homelessness (OR 5.18, 95% CI 3.17–8.46).

**Table 1:** Analysis of sociodemographic characteristics of patients admitted with RSV infection or influenza, Washington, USA, 2012–2017*

Characteristic	RSV, n = 157	Influenza, n = 708	Univariable p value	RSV vs. influenza, OR (95% CI)	Multivariable p value
Age, y (range)	56.0 (18–93)	52.8 (18–100)	0.035	1.01 (1.00–1.02)	0.01
Male sex	100 (64)	433 (61)	0.590	–	–
American Indian or Alaska Native	16 (10)	31 (4)	0.006	–	–
Black or African American	26 (17)	201 (28)	0.002	–	–
Homeless	50 (32)	137 (19)	<0.001	2.00 (1.33–3.03)	0.001
Drug use	26 (17)	60 (8)	0.005	1.79 (1.06–3.03)	0.028
Asthma/COPD	32 (20)	95 (13)	0.034	1.67 (1.06–2.63)	0.027
Smoking	38 (24)	71 (10)	<0.001	–	–

*Values are no. (%) unless otherwise indicated. COPD, chronic obstructive pulmonary disease; OR, odds ratio; RSV, respiratory syncytial virus. –, variable was excluded from multivariable analysis.

Overall, a higher proportion of adults hospitalized with RSV infection were admitted to the intensive care unit (ICU), readmitted within 30 days, and received any antimicrobial drug compared with patients hospitalized with influenza (Table 2). A total of 4% (7/158) of adults given a diagnosis of RSV infection died during hospitalization, compared with 3% (21/712) of those with influenza. Having COPD/asthma was not correlated with antimicrobial drug use (OR 1.07, 95% CI 0.71–1.60). Only 10% (4/40) of patients with RSV infection who were readmitted within 30 days had a positive swab specimen for the same virus at the second admission.

**Table 2:** Clinical characteristics of patients admitted with RSV infection or influenza, Washington, USA, 2012–2017*

Characteristic	RSV, n = 158	Influenza, n = 712	Univariable p value
Mean length of hospital stay, d	5.5	4.6	0.67
ICU admission	39 (25)	123 (17)	0.041
Mean length of ICU stay, d	3.5	3.6	0.86
Readmission within 30 d	40 (25)	79 (11)	<0.001
Patients fitting SIRS criteria at admission	78 (49)	309 (43)	0.18
Antimicrobial drugs used†	84 (53)	224 (31)	<0.001
Steroids used	22 (14)	61 (9)	0.05
Deaths	7 (4)	21 (3)	0.61

*Values are no. (%) unless otherwise noted. Four patients were admitted twice with influenza, and 1 patient was admitted twice with RSV infection. ICU, intensive care unit; RSV, respiratory syncytial virus; SIRS, systemic inflammatory response syndrome. †Included vancomycin, ceftriaxone, meropenem, azithromycin, levofloxacin, ciprofloxacin, amoxicillin, piperacillin/tazobactam, or ampicillin.

We sought to determine whether increased hospital readmission after hospitalization for RSV infection had other potential explanatory factors. We found by multivariable analysis that having RSV infection (OR 2.40, 95% CI 1.54–3.76) and homelessness (OR 2.06, 95% CI 1.31–3.24) remained associated with an increased odds of hospital readmission. Because homelessness and RSV infection increased the odds of readmission, persons at highest risk were homeless persons with RSV infection (OR 2.4 × 2.06 = 4.95 relative to housed persons with influenza). Age (OR 1.00, 95% CI 0.99–1.02), having COPD/asthma (OR 0.88, 95% CI 0.47–1.58), and drug use (OR 1.15, 95% CI 0.62–2.13) were not correlated with readmission.

We found that 6.5% (24,452/374,672) of all patients discharged from Harborview Medical Center during 2012–2017 were homeless. In that same period, 32% (50/157) of those with RSV infection were homeless, compared with 19% (147/708) of those with influenza (p = 0.003), 3.4% (286/8,488) of patients with a urinary tract infection (p<0.001), and 2.0% (25/1,278) of patients with an ischemic stroke (p<0.001).

## Conclusions

In this study of adults hospitalized during 5 years in an urban hospital, 32% of patients given a diagnosis of RSV infection were homeless, compared with 6.5% of all patients hospitalized. Patients hospitalized with RSV infection were more likely to be older, homeless, drug users, or have COPD/asthma compared with persons with influenza. Homelessness has reached a national public health crisis, and many homeless persons seek acute care in emergency departments (*9*). The city of Seattle has the largest concentration of homelessness per capita in the country (*10*). Outbreaks of infections with respiratory viruses have been described in homeless shelters, in which transmission might be facilitated by crowding, poor sanitation, and the ability of RSV to spread through fomites (*6*,*11*). Our findings for RSV in this homeless population might be generalizable to other urban public hospitals.

Several outcome measures were worse in patients hospitalized for RSV infection than for influenza, including 30-day readmission, admission to the ICU, and receipt of antimicrobial drugs. A previous study similarly reported higher rates of ICU admission among patients with RSV infection than for those with influenza (*12*). More severe disease might have led clinicians to preferentially use antimicrobial drugs for patients hospitalized with RSV infection compared with influenza. The higher number of patients with RSV infection than influenza admitted to the ICU supports this interpretation. These results suggest that patients hospitalized with RSV infection might benefit from closer monitoring, follow-up, and antimicrobial drug stewardship to prevent readmission and overuse of antimicrobial drugs.

Homelessness and having RSV infection were independent risk factors for hospital readmission, demonstrating that the higher risk for poor outcomes in homeless persons was not simply explained by the disproportionately higher number of diagnosis of RSV infection in this group. All-cause readmission within 30 days is a major quality metric used by the Centers for Medicare and Medicaid Services and Hospital Quality Alliance (http://www.allhealthpolicy.org/glossary/hospital-quality-alliance). Other studies have found an association between lower education and unemployment with rehospitalization and that most rehospitalizations were related to concurrent conditions (*13*).

Limitations of this study include the retrospective study design, limiting analysis to only hospitalized patients with RSV infection or influenza. The overall number of hospitalizations annually for RSV infection and influenza increased during 2012–2017 and was likely caused by transition to on-site rapid testing, which might increase provider uptake (*14*). In addition, without hospitalized and community controls who do not have influenza or RSV infection and who are not homeless, we cannot definitively conclude that homelessness is associated with a greater risk for hospitalization for RSV infection compared with influenza. The association might have 3 possible interpretations: risk factors are associated with more severe disease caused by RSV than influenza; risk factors are associated with a higher risk for infection with RSV compared with influenza; or risk factors are associated with greater susceptibility to RSV infection and disease compared with influenza. In addition, this study was limited to a single site, although it is representative of public, safety-net hospitals. Additional limitations include clinician-initiated testing triggered by influenza-like symptoms rather than for detection of RSV infection, which is less likely to manifest with fever (*15*). Therefore, the true burden of RSV infection is likely higher than identified in this study.

In conclusion, homeless persons might represent a previously unrecognized population at increased risk for poor outcomes caused by infection with RSV. An effective vaccine or therapeutic in adults could benefit this medically underserved population. Further data on the impact of homelessness on respiratory virus infection severity and outcomes are needed to guide public health strategies and implementation.

Appendix Additional information on respiratory syncytial virus infection in homeless population, Washington, USA.
