# Supplementary material for: Respiratory Syncytial Virus Infection in Homeless Populations, Washington, USA
Source: Emerg Infect Dis. 2019 Jul;25(7):1408–11. doi: 10.3201/eid2507.181261 (PMC6590761; doi:10.3201/eid2507.181261)
Supplement: Appendix — Additional information on respiratory syncytial virus infection in homeless population, Washington, USA. [file 18-1261-Techapp-s1.pdf]

Article DOI: https://doi.org/10.3201/eid2507.181261

Respiratory Syncytial Virus Infection in Homeless Populations, Washington, USA

Appendix

Methods

Patient Population

Clinical and laboratory data was extracted from the electronic medical record through the Enterprise Data Warehouse (Inspirata, https://www.inspirata.com/idap-nlp-as-a-service) and entered into Project Research Electronic Data Capture. Homelessness was defined as an International Classification of Diseases, 10th revision diagnosis of homelessness, presence of the term "homeless," "staying with friends," "living in a hotel/motel/shelter/tent/car," or "living on the streets" in the admission note or patient registration. Drug use was defined by a history of cocaine, heroin, crack, speed, or recreational marijuana use, or by a patient answering "yes" to the question on the admission form asking, "Do you or have you taken illicit drugs?"

The overall number of discharges, admissions with a diagnosis of a urinary tract infection, ischemic stroke, and cellulitis at Harborview Medical Center (Seattle, WA, USA) was collected during July 1, 2012–June 30, 2017. Severe illness was defined as hospitalization in the intensive care unit. Systemic inflammatory response syndrome was defined by $\geq$2 of the following: leukocyte count >12,000 cells/mm^3 or <4,000 cells/mm^3, heart rate >90 beats/min, respiratory rate >20 breaths/min, and temperature >38°C or <36°C. Readmission was defined by a second inpatient admission to Harborview Medical Center within 30 days of discharge from the initial admission.

Statistical Analysis

Data analysis was performed by using Stata version 15.0 (StataCorp LLC, https://www.stata.com/company). Univariable proportions were compared by using χ^2 and Fisher exact tests. Risk for hospitalization with respiratory syncytial virus infection versus influenza was evaluated by using multivariable logistic regression analysis. We decided a priori to include

statistically significant variables from univariable analysis of hospital admissions with respiratory syncytial virus versus influenza (age, homelessness, having chronic obstructive pulmonary disease or asthma, and drug use) in multivariable regressions. Smoking was not separately included because smoking can be correlated with chronic obstructive pulmonary disease. Backward elimination was used to exclude variables that were not meaningfully predictive of outcomes. A P value <0.05 was considered statistically significant. The study was approved by the institutional review board of the University of Washington.
